# Alveolar macrophages in tissue homeostasis, inflammation, and infection: evolving concepts of therapeutic targeting

**DOI:** 10.1172/JCI170501

**Published:** 2023-10-02

**Authors:** Christina Malainou, Shifaa M. Abdin, Nico Lachmann, Ulrich Matt, Susanne Herold

**Affiliations:** 1 Department of Internal Medicine V, Universities of Giessen and Marburg Lung Center, Justus Liebig University Giessen, Member of the German Center for Lung Research (DZL), Giessen, Germany.; 2 Institute for Lung Health, Justus Liebig University Giessen, Giessen, Germany.; 3 Excellence Cluster Cardio-Pulmonary Institute, Giessen, Germany.; 4 German Center for Lung Research (DZL), Heidelberg, Germany.; 5 Department of Pediatric Pneumology, Allergology and Neonatology and; 6 REBIRTH Center for Translational and Regenerative Medicine, Hannover Medical School, Hannover, Germany.; 7 Fraunhofer Institute for Toxicology and Experimental Medicine, Hannover, Germany.; 8 RESIST (Resolving Infection Susceptibility), Cluster of Excellence, Hannover Medical School, Hannover, Germany.

## Abstract

Alveolar macrophages (AMs) are the sentinel cells of the alveolar space, maintaining homeostasis, fending off pathogens, and controlling lung inflammation. During acute lung injury, AMs orchestrate the initiation and resolution of inflammation in order to ultimately restore homeostasis. This central role in acute lung inflammation makes AMs attractive targets for therapeutic interventions. Single-cell RNA-Seq and spatial omics approaches, together with methodological advances such as the generation of human macrophages from pluripotent stem cells, have increased understanding of the ontogeny, function, and plasticity of AMs during infectious and sterile lung inflammation, which could move the field closer to clinical application. However, proresolution phenotypes might conflict with proinflammatory and antibacterial responses. Therefore, therapeutic targeting of AMs at vulnerable time points over the course of infectious lung injury might harbor the risk of serious side effects, such as loss of antibacterial host defense capacity. Thus, the identification of key signaling hubs that determine functional fate decisions in AMs is of the utmost importance to harness their therapeutic potential.

## Introduction

The pulmonary macrophage pool reflects a heterogeneous population of cells, determined by anatomical niche occupancy and their history of exposure to pathogens, and is characterized by high functional plasticity. In addition to macrophage precursors mobilized from the bone marrow in acute and chronic inflammation, diverse subsets of tissue-resident macrophages (TRMs) occupy defined niches in lung tissue. Interstitial macrophages reside in the interstitial space close to the bronchovascular bundle (and, rarely, in the alveolar interstitium) (1), adjacent to neurons, lymphatics, endothelium, and other hematopoietic cells (2). Alveolar macrophages (AMs) constitute the main immune cell population of the alveolar airspace during homeostasis and serve as gatekeepers of intact immunity, endowed with cytosolic and membrane receptors allowing for a “high-alert” state regarding any invading pathogens (3–9). Though not traditionally seen as targets for therapeutic strategies aiming at reshaping the immune response, AMs possess various characteristics that might render them interesting candidates for therapeutic approaches. Strategically placed within the alveoli, these professional phagocytes serve as intermediates between the outside world and the organism. This positioning shapes a unique transcriptomic signature, which combines resident macrophage (10, 11) and lung-specific gene expression (12).

Under homeostatic conditions, clearance of pulmonary surfactant and cellular debris is the main task of AMs (3, 13, 14). Though not every alveolus contains an AM, it has been proposed that AMs are capable of migrating through adjacent alveoli using the pores of Kohn to patrol the airspaces and clear pathogens without activation of a broad immune response, yet the majority of them are considered sessile cells in the absence of injurious stimuli (15–17). AMs exert trophic functions aimed at the epithelium through the release of growth factors such as TGF-β, regulate fluid transport, and induce epithelial cell proliferation (18–20). Direct AM contact with the alveolar epithelium through connexin 43–containing gap junctions using the CD200/CD200R signaling axis acts as an immunosuppressive signal during homeostasis and thwarts unwanted immune responses (17, 21). Additionally, in steady-state conditions, surfactant proteins A and D suppress the phagocytic capacity of AMs by binding to surface-expressed signal-regulatory protein α (SIRPα) (21, 22). AMs also prevent aberrant activation of resident T lymphocytes as a response to antigen presentation by dendritic cells (23–25). The lung microbiome is a further determinant of AM activation state. While a “balanced” microbiome promotes immunotolerance, microbiota dysbiosis can activate catabolic pathways and alter AM functions (26, 27). Thus, depending on the local microenvironment conditions, their functional polarization, and the qualitative and quantitative challenge, AMs are able to maintain tissue homeostasis, by preventing infections and aberrant immune responses. Should pathogen containment fail, AMs can initiate a well-orchestrated inflammatory response, while retaining an important role as drivers of resolution of inflammation in the aftermath of infection.

Development and maintenance of AMs rely on GM-CSF and TGF-β, which activate the transcription factor PPARγ (5, 28, 29). Originating in the yolk sac, AM precursors seed the lung during the late embryogenesis phase and continue differentiating during early life (30). TRM functions seem to be dictated by origin, differentiation state of the precursor, tissue of residence, inflammation experience, and time spent in a specific environment (7–9, 11, 31–34). Unlike TRM populations that reside in a niche easily accessible to vasculature (e.g., liver, epidermis), AMs are located in a microenvironment that is secluded from circulation during homeostasis (6, 7, 28). This might explain the capacity of AMs for self-renewal, which is adequate for resolving minor losses without any major contribution from the periphery. Upon severe acute and/or chronic inflammation, however, macrophage precursors are mobilized from the bone marrow, creating a diverse lung TRM niche over time (4). As an example, elegant work has shown that bone marrow–derived AMs exhibit different functions in the aftermath of viral infections (34, 35). However, the persistence of these alterations and the contribution of environmental cues as opposed to ontogeny for functional differences are difficult to disentangle.

State-of-the-art technologies, such as single-cell RNA-Seq/CITE-Seq analyses, spatial omics, and fate mapping, have revealed extensive knowledge regarding TRM ontogeny (9, 36–38), macrophage polarization profiles (14, 39, 40), and the therapeutic potential of immunomodulatory treatments aimed at shifting the balance between the differently polarized profiles of AMs (41, 42). Below, we discuss the role of AMs in infection-associated lung diseases, current models of translational AM research, and the impact of AMs on novel immunotherapy discovery, while presenting AMs as a therapeutic target in pulmonary medicine.

## The diverse role of AMs in infection-related and inflammatory lung diseases

### AMs as central regulators of the innate immune defense in respiratory diseases.

The tolerogenic programming of AMs is overrun upon infection, when inertly antiinflammatory AMs overcome local immunosuppressive signals and switch to a more proinflammatory phenotype (20, 43). Given the plasticity of AMs and their spatial distribution in the lung, AMs play indispensable and quite versatile roles responding to the inhaled pathogens they are faced with.

AMs can initiate leukocyte recruitment (44, 45) and directly eliminate the pathogen using multiple pathogen-specific mechanisms (15). For example, upon encounter of *Legionella pneumophila*, AMs activate the interleukin-1β (IL-1β) pathway to recruit neighboring monocytes (46). Upon challenge with *Aspergillus fumigatus*, murine AMs control infection by activating the NADPH oxidase and ROS pathway (47, 48). When the overall bacterial load exceeds their capacity for pathogen degradation in the phagolysosomes, as observed with *Streptococcus pneumoniae*, AMs can switch on the apoptosis pathway in order to prevent bacterial dissemination, through the classical process of phagocytosis, as observed in a murine infection model (49). Well-adapted pathogens like *Mycobacterium tuberculosis* are capable of subverting macrophage containment strategies to ultimately promote their dissemination (50).

AMs are critical coordinators of the immune response against viral pathogens, via secretion of proinflammatory cytokines/chemokines such as IL-6, IL-8, or CXCL10, initiation of type I interferon (IFN) signaling, and enhanced expression of pattern recognition receptors, as well as inhibition of nuclear export of viral genome as shown in a series of elegant studies on influenza virus infection (51–55). Further murine infection models also revealed that AMs can utilize other molecular mechanisms, such as arginase-1 expression, phagocytosis of fungal conidia (48, 56), directed migration toward and phagocytosis of bacterial pathogens, and release of IFN-γ (15, 57), to support pathogen clearance from the alveoli, which is eventually performed by recruited neutrophils and bone marrow–derived macrophages (BMDMs). Depletion of the AM pool, either experimentally (e.g., by clodronate) or during the natural course of disease (often observed in respiratory viral infection) (58–60), is linked to increased morbidity and mortality (61–67). Increased AM death and reduced self-renewal capacity (68) might account for this, yet the molecular mechanisms of AM depletion are not fully elucidated.

Depending on the type of injury and the extent of AM loss, the alveolar niche can be replenished either through local proliferation or through the recruitment of BMDMs from the periphery, as a response to chemoattractants such as CCL2 (69). These newly recruited macrophages exhibit several transcriptional differences from their resident alveolar counterparts regarding metabolism, proliferation, and inflammation signaling (70, 71). AMs are capable of self-renewal and mainly use the tricarboxylic acid cycle and amino acid metabolism in order to maintain their functions, whereas recruited BMDMs are governed by glycolysis and arginine metabolism, while adopting a more proinflammatory profile (66, 70, 72). This profile is characterized by the abundant release of molecules such as TNF-α, IL-6, and inducible NOS, which are instrumental for pathogen clearance during the acute infection phase (53, 73). As opposed to that, BMDMs can also drive inflammatory tissue damage characterized by epithelial injury, degradation of extracellular matrix, and cytokine-dependent prolonged inflammation, which aggravate barrier loss and gas exchange function and may worsen disease outcome (60, 64, 66, 73–79). Addressing specific targets related to the proinflammatory response of these cells could, therefore, mitigate their injurious potential without compromising host defense and pathogen clearance. In this regard, blocking of macrophage-derived TRAIL attenuated epithelial cell apoptosis and improved edema reabsorption and survival following severe influenza A virus infection in mice (73, 76), while membrane-tethered matrix metalloprotease (MT1-MMP) inhibition attenuated tissue damage without altering the immune response (79).

Generally, various types of injury over an individual’s lifespan result in the gradual replacement of the original, yolk sac–derived AMs by BMDMs (80), creating a mosaic of resident macrophages of different ontogeny, with distinct transcriptome signature, metabolic profile, and responses to infection (81). This heterogeneous constitution must be taken into account in considering macrophage-based therapeutic interventions in pneumonia or lung injury. Given that BMDM-replenished resident AMs may retain defined lineage- or inflammation-imprinted BMDM characteristics (35, 66), it is of utmost importance to pinpoint the correct timing and definition of the functional macrophage phenotype required to be targeted or promoted in a specific type of injury.

### AMs as drivers of inflammation resolution.

AMs actively contribute to inflammation resolution and to the repair of the denuded airways through secretion of immunomodulatory cytokines such as IL-10, CCL22, and a plethora of growth factors, including TGF-β, VEGF, trefoil factor 2, and PDGF (35, 82–84). However, alveolar cues that initiate the resolving properties of AMs are largely unknown. Interestingly, TNF-α released by AMs was shown to promote the release of GM-CSF by alveolar epithelial cells and thus support alveolar epithelial repair (85). In a model of helminth infection, IL-4 and IL-13, together with apoptotic cells, upregulated antiinflammatory and tissue-repair genes within AMs (86). Genetic deletion of AXL and MERTK, which are crucial receptors for phagocytic uptake, impaired resolution of inflammation in this model, underlining the importance of efferocytosis.

Repair processes following injury require a tight balance between inflammation resolution, epithelial barrier restoration, and the prevention of aberrant remodeling. Newly replenished, pro-regenerative AMs exhibit a profibrotic potential in animal models of bleomycin-induced fibrosis, in patients with pulmonary fibrosis, and during infection (87–91). In the context of COVID-19, human monocyte-derived AM subsets contribute to immunopathology by perpetuating inflammation and promoting post-resolution profibrotic pathways, leading to aberrant lung remodeling and an idiopathic pulmonary fibrosis–like phenotype (92–94). The specific mechanisms involved remain a matter of intensive research, aimed at dissecting the intertwined roles of innate and adaptive immunity in these processes (60, 78, 94). Reprogramming of AMs and targeting of fibrosis-related intracellular pathways in favor of a coordinated and accelerated epithelial repair, therefore, emerge as potential therapeutic strategies for a clinical phenotype with remarkably limited treatment options (90, 95, 96).

Aging adds an additional level of complexity to the aforementioned differentiation trajectories and AM phenotype. The transcription profile of AMs heavily depends on age, with cell cycle–related genes being downregulated in aging animals and humans (61, 81). Upregulation of genes related to injury resolution and lung remodeling is a characteristic of resident AMs found in the lungs of aged mice (97). Local microenvironment is an important factor shaping AM functions with age (66, 97). Hyaluronan increase in the extracellular matrix of the lungs of aged animals diminishes the pro-proliferative effect of GM-CSF on macrophages, which could explain the gradual decrease in AM numbers over time (81). Aging additionally impairs the phagocytosis and efferocytosis capacity of AMs, leading to retention of activated neutrophils within the alveoli with prolonged lung damage in mice (61). Adoptive transfer of rejuvenated AMs into an aged lung could, therefore, constitute a potential therapeutic intervention.

### Pneumonia results in pathogen- and injury-dependent reprogramming of AM functions.

Inflammation-derived lung injury is characterized by enhanced susceptibility to secondary bacterial hits in the aftermath of infection (98–101) or acid pneumonitis (102). This susceptibility can primarily be attributed to diminished numbers, impaired bactericidal properties, and the adoption of a pro-resolution phenotype by AMs (45, 58, 102–107). Human and murine infection studies showed that as inflammation subsides, T lymphocytes orchestrate an IFN-γ–driven downregulation of macrophage receptor with collagenous structure (MARCO) expression on AMs (82, 108–110), diminishing antibacterial properties of AMs (111). Depletion of IFN-γ was, therefore, shown to restore bacterial control in this particular context (110). AMs also actively terminate neutrophil influx in murine models of bacterial and LPS-induced inflammation (112–114). NO production is reduced, while the 15-lipoxygenase pathway, which is initiated by AMs during efferocytosis, further promotes inflammation resolution (115, 116). However, this shift toward an antiinflammatory phenotype comes at the cost of bacterial clearance (117, 118). Medeiros et al. delineated in an animal study that prostaglandin E2 (PGE2) dampens bacterial clearance (118). In vitro, pharmacological interference with this pathway restored antibacterial control. Notably, PGE_2_ inhibition in influenza virus–infected mice enhanced immunity against infection (119), illustrating the impact of immunomodulation during acute lung injury.

Efferocytosis is a key event of inflammation resolution and the initiation of tissue repair (120–122). Macrophages are capable of responding to “find me” (123, 124) and “eat me” signals from apoptotic cells through a variety of scavenger or tyrosine kinase receptors, integrins, and complement receptors (125–127). Intriguingly, cell type–specific efferocytosis of neutrophils rewires mitochondrial metabolism to switch AMs to a pro-resolution phenotype. Neutrophil myeloperoxidase locked AMs in a state of pro-resolution, which precluded generation of mitochondrial ROS via uncoupling protein 2 upon bacterial encounter (128). Roquilly et al. described SIRPα-dependent impaired antibacterial properties in AMs months after bacterial pneumonia, conferred by the lung environment (129). AMs from SIRPα-knockout mice maintained phagocytic functions, and peripheral blood monocytes of patients exhibited improved phagocytosis after treatment with an anti-SIRPα antibody (129). In contrast, depending on the specific infection context, reprogramming of AMs may result in improved bactericidal properties, as a sign of trained immunity. Prior mycobacterial infection or adenovirus exposure improved AM defense against a secondary bacterial infection (130, 131), or resulted in heterologous protection against a rechallenge with *M*. *tuberculosis* (132). Guillon et al. revealed that after *S*. *pneumoniae–*induced pneumonia in mice, the newly acquired AM phenotype was long-lasting over a period of 6 months, regionally localized to the affected lung lobe, and resulted in enhanced AM-driven protection against another pneumococcal serotype (133).

## Translational models of AMs and impact on immunotherapy

### Ex vivo culture models of murine AMs.

Knowing the vital roles of AMs in regulating lung homeostasis and inflammation necessitates better understanding of the underlying mechanisms pertaining to AM functions. Although immortalized murine AM cell lines, such as MH-S cells, exist, these cells lack key hallmarks of AMs, such as expression of sialic acid–binding Ig-like lectin-F (Siglec-F), and the specific phenotype imprinted by the lung environment (134, 135). Studies using primary AMs have improved our knowledge of AM biology. However, lavage-isolated primary AMs do not reflect the whole AM pool, as AMs that are more adherent to the epithelium might be missed (136).

**Figure 1:**
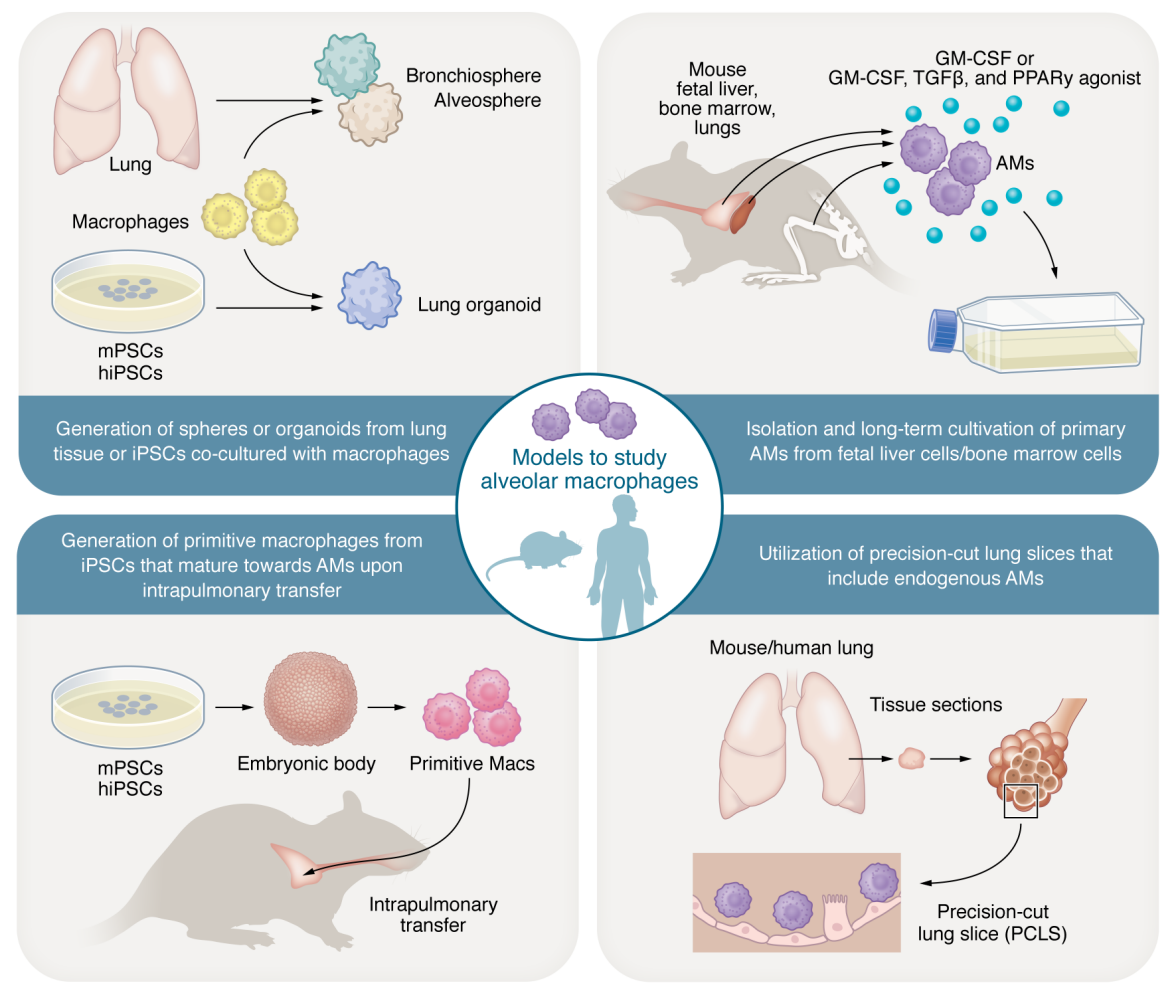
Depiction of diverse murine/human-based AM culturing models promoting translational AM research. The figure summarizes the various modern ex vivo culturing approaches that have used murine or human-derived AMs in combination with human induced pluripotent stem cells (hiPSCs) or murine pluripotent stem cells (mPSCs), mediating the transition to 3D culturing systems (organoids, precision-cut lung slices, etc.), all dedicated to studying the biology of AMs and potential therapies targeting these cells.

Recently, several groups investigated different cell culture models to replicate the AM phenotype and functions in ex vivo settings (Figure 1). Murine fetal liver cells can serve as a resource for the long-term culture of self-replicating AMs. Culturing fetal liver cells with GM-CSF gave rise to self-replicating AMs that could be sustained in culture for 8 weeks (137). Long-term culture of non-transformed AMs was also achieved from other cell sources, such as adult bone marrow cells (138). The synergistic effect of GM-CSF in combination with TGF-β and PPARγ agonist such as rosiglitazone was essential to reproduce the AM-like phenotype from cultured adult bone marrow cells. The generated AM-like cells from this culturing condition expressed typical AM surface markers (Siglec-F, CD11c), AM-specific genes such as carbonic anhydrase 4 (Car4), placenta-expressed transcript 1 (Plet1), and self-renewal genes (138). The triple combination of these growth factors resulted in prolonged expansion of mouse ex vivo–cultured AMs (mexAMs) over several months and similar functionality to primary AMs in terms of phagocytosis capacity and cytokine secretion upon challenge with *S*. *pneumoniae* or LPS (139). Intranasal transfer of mexAMs showed successful transfer, engraftment, and proliferation of the administered cells. Similarly, adoptive transfer of mexAMs into mice lacking AMs because of GM-CSF deficiency resulted in the restoration of lung homeostasis (139).

Such optimized murine AM models provide powerful tools to deepen our understanding of AM biology and behavior during homeostasis and disease. Nevertheless, single-cell RNA-Seq experiments strongly indicate that the lung AM populations are heterogeneous (6, 140). This heterogeneity might not be ideally reflected in models in which mice were maintained under tightly controlled, specific pathogen–free conditions, with limited exposure to lung insults, and breeding facility–specific microbiome that might substantially influence AM phenotype and function (141, 142). AMs in the lung niche of naive laboratory mice are mostly of embryonic origin. In contrast, the AM pool in humans is a mix of embryonic and BMDM-derived AMs (143, 144). Moreover, the lung macrophage compartment is much more diverse in humans, compared with mice, at least in the context of lung cancer (144). In fact, transcriptomic analysis revealed proliferating and non-proliferating AMs in healthy humans, in addition to the added diversity through the characterization of newly emerging AM subtypes within different disease entities, such as fibrosis and malignant lung disease (145).

### Ex vivo culture models of human AMs.

To overcome the shortcomings of murine-based systems and to provide a more clinically relevant model of AM culture, several approaches have been taken to either prolong the primary culture of AMs or identify a regenerative supply of macrophages from different stem cell sources. In this context, a technique was developed to culture human AMs ex vivo from resected lung tissues of patients with pulmonary tuberculosis, caused by *M*. *tuberculosis* (146). This method revealed important insights into the interaction between *M*. *tuberculosis* and human cells, identifying factors that can control mycobacterium infection in the lung microenvironment (147).

Recently, Pahari et al. demonstrated that human blood-derived monocytes cultured with lung lipids, GM-CSF, TGF-β, and IL-10 exhibited similar morphology, transcriptional profile, and functions to those of human AMs (148). While the ability to culture primary cells ex vivo is helpful, such approaches are limited by the lack of a three-dimensional environment that can mimic the cell-to-cell interaction and recapitulate the complex cellularity of the lung parenchyma and airways. Hence, other 3D ex vivo systems should be explored, such as spatially organized multicellular human lung organoids or human precision-cut lung slices (hPCLSs). PCLSs are slices of healthy or diseased lungs containing all cell types found in the desired tissue, while still mirroring the underlying changes in the context of different diseases affecting the extracellular matrix. Use of hPCLSs revealed important insights into the ability of *Staphylococcus aureus* to survive and grow in AMs. *S*. *aureus* cannot use AMs for intracellular growth during infection but is, rather, present in the epithelial and interstitial regions of hPCLSs, highlighting important antibacterial features of AMs in a preserved human lung environment (149).

While isolation of primary AMs from human samples is clinically relevant, it is also hampered by low yields and the inability to genetically engineer these cells for disease modeling. In addition, isolation techniques can have detrimental impacts on the quality and marker expression patterns of the isolated AMs, necessitating caution in interpreting the results. Along these lines, human induced pluripotent stem cells (hiPSCs) can meet the need for scalable, personalized, and standardized sources of human macrophages. Additionally, iPSCs have the advantage of being easily manipulated by genome editing methods (CRISPR/Cas9, TALENs), providing a powerful tool for disease modeling (150). iPSC-derived macrophages (iMacs) share the phenotypic, functional, and transcriptional hallmarks of professional phagocytes, while the transcriptional profile resembles that of primitive macrophages (151–153). In fact, iPSCs are thought to drive macrophage differentiation predominantly through the embryonic hematopoiesis pathway, in a MYB-independent but RUNX1- and SPI1 (PU.1)–dependent fashion. Correspondingly, TRMs, including AMs, are of embryonic origin, driven from their yolk sac progenitors in a MYB-independent fashion. This suggests that iMacs represent better models of TRMs compared with primary monocytes (153, 154).

Another advantage to the use of iMacs is the capacity to scale up iMac generation, allowing long-term production of industrial quantities of iMacs with consistent quality. iMacs can also be incorporated into 3D culture systems that can feature the human “lung in a dish.” Research efforts in human lung organoids resulted in major advancements in identifying the molecular cues needed to facilitate the establishment of various models with different spatial complexity and levels of epithelial cell commitment and differentiation. Currently, lung organoids stemming from adult human lung-committed progenitor cells such as basal cells or alveolar type II cells (giving rise to bronchiospheres and alveolospheres), from fetal lung progenitor cells, or from iPSCs can harbor multiple airway and alveolar cell types and are used in either 3D culture or in vivo transplantation models (155–158). iMacs can be successfully incorporated into lung organoids stemming from iPSCs, providing an indispensable tool to study the interaction of macrophages with the human lung environment in homeostasis and disease, including infection, while overcoming many of the limitations of in vivo animal models or ex vivo–derived murine and human primary cultures (Figure 1). In fact, incorporating iMacs into alveolar organoids resulted in higher levels of chemokines after LPS stimulation (159). Similarly, murine lung organoids complemented with AMs had higher chemokine and cytokine levels than organoids without AMs (160). iPSC-derived, iMac-complemented lung organoids harbor the additional advantage of generating patient-specific models for future personalized diagnostic or treatment approaches (161).

With the continuous advancement of currently existing translational AM models and the knowledge gained on AM biology, these cells are currently emerging as highly promising candidates for the treatment of pathogen-driven inflammatory lung diseases.

## AMs as emerging therapeutic tools in lung inflammatory disease

### AMs as a cell-based therapeutic regimen.

The regenerative feature of the pluripotent stem cell (PSC) system and the convenience of generating murine or human macrophages in upscaled quantities under good manufacturing practice conditions in a continuous fashion have promoted the application of AMs as a cell-based therapeutic option.

Several groups have examined the potential of macrophage transplant therapy in resolving different pulmonary diseases. In one study, AMs derived from murine PSCs were used to ameliorate acute and chronic lung injury. To mimic the acute lung injury (ALI) model, AMs were partially depleted, whereas the chronic lung injury was modeled using adenosine deaminase (ADA) knockout mice that typically suffer from a plethora of pulmonary abnormalities with pulmonary failure and poor survival. The intratracheal administration of PSC-AMs was a successful and safe strategy to resolve the acute and chronic injury by engulfing neutrophils and driving lung repair, thus prolonging survival, while AMs persisted in the airways for a minimum of 4 weeks (162).

In cases in which the hyperinflammatory environment is the main driver of ALI, the therapeutic intervention would vary, requiring the neutralization of the inflammatory environment instead of merely boosting of the AM niche. Hence, in an ALI model induced by LPS administration, skewing of the AM phenotype to an antiinflammatory profile was examined. This was achieved by pulmonary macrophage transfer of murine engineered macrophages (RAW264.7 macrophages or BMDMs) that can secrete IL-4. The sustained secretion of IL-4 from AMs polarized the macrophages to a pro-resolution phenotype, halted the harmful proinflammatory cascade, and activated tissue repair and remodeling programs (163).

Beyond the use of AMs as a cellular therapy for lung tissue repair and homeostasis maintenance, adoptive transfer of AMs was also a successful strategy in combating pulmonary infections. In proof-of-concept studies, adoptive transfer of iMacs successfully rescued mice from *Pseudomonas aeruginosa*–mediated acute infections within 4–8 hours after intrapulmonary transplantation, with a reduction of the bacterial load (151). Similar findings were also provided by a murine pneumonia model of methicillin-resistant *S*. *aureus* (MRSA), where the transplanted iMacs were able to elicit a more than 10-fold reduction of the bacterial load within 20 hours of adoptive transfer (164).

Overall, these data highlight the antiinflammatory/pro-repair properties of AMs. However, proinflammatory functions have to be taken into account and deserve further studies, particularly in the setting of ALI, where inflammatory tissue damage determines outcome.

### Correction of dysfunctional AM phenotypes.

Cell-based therapy is of particular importance in individuals suffering from genetic diseases that cause malfunctioning AMs. Hematopoietic stem cell transplantation (HSCT) has been studied in that regard as a non-cell-specific strategy to correct deficiencies in the AM compartment. Cystic fibrosis (CF) is one prominent example of a genetic disease–causing mutation in the CF transmembrane conductance regulator (CFTR) protein, which ultimately leads to defective mucus clearance and heavy bacterial colonization of the lung by various species that develop antibiotic resistance. In addition, loss of CFTR in macrophages impacts proper phagosome acidification (165), and macrophages derived from CF patient–specific iPSC lines exhibit a dysregulated type I IFN response (166). Hence, in such a devastating disease course, healthy AMs may be key players in controlling the associated pulmonary infections.

HSCT was performed in CFTR-deficient mice suffering from acute airway infection with *P*. *aeruginosa*. The transfer of competent stem cells allowed the differentiation and successful homing of healthy AMs that cleared the bacterial infection (167). Another in vivo murine study demonstrated the promising potential of this intervention in the context of the Mendelian susceptibility to mycobacterial disease (MSMD) syndrome. Genetically impaired IFN-γ–mediated immunity characterizes this syndrome, manifesting within the lymphoid and myeloid compartment, and limits tissue macrophage capacity to clear mycobacteria. Gene-corrected hematopoietic stem cells that were transplanted into *Ifnγr1^–/–^* mice infected with *Mycobacterium bovis*, Bacille Calmette-Guérin (BCG), gave rise to AMs with restored antibacterial activity against BCG, maintained lung integrity, and prolonged overall mouse survival (168).

AM-specific targeting approaches were used to investigate whether primary macrophages could be directly engineered to correct a pathological phenotype. To demonstrate the feasibility of this approach, a third-generation self-inactivating lentivirus was used to engineer primary human and murine macrophages inducing the expression of the human α_1_-antitrypsin (hAAT). Such macrophages could be used to compensate for AAT deficiency in affected individuals suffering from pulmonary emphysema as a consequence of the deficiency. Overexpression of hAAT in murine AMs (an AM cell line and BMDM-derived AMs) resulted in secretion of hAAT in vivo, which was detected in the bronchoalveolar lavage fluid of AM-transplanted mice. Secretion of hAAT by the genetically corrected administered macrophages also correlated with increased overall survival after BCG infection, suggesting that genetic engineering of primary monocytes/macrophages could be a potentially valid approach to address pulmonary disease (169).

Hereditary pulmonary alveolar proteinosis (PAP) is another genetic disease that can benefit from AM-based cellular therapy. In this disease, recessive mutations in the GM-CSF receptor genes CSF2RA and CSF2RB lead to a subsequent defect in the GM-CSF receptor, which correlates with poor AM differentiation and function. Clinically, this is associated with a predisposition to respiratory infection and lung failure (170). Intratracheal application of iMacs in a humanized model of hereditary PAP showed promising therapeutic potential. Two months after engraftment, iMacs differentiated into functional AMs, reducing the surfactant levels and restoring lung homeostasis (170). Similarly, modeling hereditary PAP using *Csf2ra-*knockout mice revealed that the pulmonary transfer of genetically corrected macrophages expressing GM-CSF receptor α can improve the disease phenotype and mediate long-term AM engraftment lasting up to 6 months (171). While the aforementioned studies highlight the promising potential of adoptive macrophage transfer for cell-based therapy, many factors need to be determined to guarantee the successful clinical translation of such an approach. One of these factors is the durability of the administered treatment, as different studies report varying findings concerning how long transplanted AMs are sustained in the lung niche, with the longest reported period reaching up to 3 years after transplant (172).

Donor tolerance to the allograft is a further key determinant of cell transplantation success. Preconditioning of the lung environment prior to macrophage transplant using a liposomal macrophage-depleting agent like clodronate was shown to promote hematopoietic chimerism, resulting in better donor tolerance to the received cells (173). The possible occurrence of graft-versus-host disease upon the subsequent transfer of allogenic AMs must, however, be fully addressed before ex vivo findings can be transferred to the clinic. Current knowledge on this subject is limited, in particular regarding the extent of the immune reaction that such a transplantation therapy may elicit as well as the ideal immunosuppressive method. For instance, MHC I–mismatched AMs are able to elicit humoral and cellular immune responses to lung-associated self-antigens (172).

### AMs as targets for drug delivery and for modification of their functional state.

Several features facilitate therapeutic targeting of AMs in the lungs. AMs are positioned at an easily accessible site, allowing administration of drugs by nebulization techniques that generate small droplet sizes to reach the alveolar compartment, or targeting by drug-containing particles or other carriers.

Novel formulation techniques were used to promote AM-targeted antibiotic therapy. In a recent study, the authors used a polymeric ciprofloxacin prodrug with a linker for mannose ligands. The abundant expression of mannose receptors on the surface of AMs facilitated ciprofloxacin intake by AMs, and the formulation was more effective in protecting the mice from a *Burkholderia* *pseudomallei* clinical isolate as compared with the administration of the free drug (174). Furthermore, liposome-based formulations were very successful for AM-targeted antibiotic therapy. Liposomal amikacin was recently approved by the FDA for treatment of non-tuberculous mycobacterial biofilms with high efficacy of the antibiotic in the infected AMs (175). Lipid nanocarriers encapsulating levofloxacin were conjugated with fucosyl residues, which enabled the targeting of the mannose receptor–positive AMs. As in other studies, the encapsulated antibiotic showed superior activity compared with the “free” antibiotic in the mycobacterial clearance of infected AMs. Likewise, ciprofloxacin in liposomal formulation showed protective effects against pneumonic plague in vivo (176).

Other AM-targeting therapeutic strategies have aimed at activating different cellular programs (pro- or antiinflammatory) of AMs based on the needed role in the given disease state. For example, the activation of PPARγ controlled host response and lung pathology during influenza virus pneumonia in mice by skewing AM polarization status toward the anti–inflammatory macrophage subtype (177). Similar findings were seen in a bleomycin-induced lung injury model, where PPARγ activation was linked to an enhanced AM efferocytic potential and a tight regulation of IL-10 and TGF-β secretion.

The functional (re)polarization of AMs is of particular importance in infectious and inflammatory lung injury, due to the critical role of macrophages in all stages of the disease. Recent efforts have focused on repurposing drugs for inflammatory lung injury and infections to switch the proinflammatory state of AMs to an antiinflammatory, pro-resolution phenotype, or to increase macrophage host defense, as reviewed in ref. 178. Zanubrutinib targets Bruton’s tyrosine kinase (BTK) activation, inhibiting JAK2/STAT1 and TLR4/MyD88/NF-κB signaling pathways and promoting activation of STAT6 and PI3K/Akt signaling pathways (179). Canagliflozin is an SGLT2 inhibitor with pronounced antiinflammatory actions that inhibits the IL-1β–stimulated secretion of IL-6 and impacts glucose metabolism in macrophages (180). As these strategies were applied in sterile lung inflammation models, the impact of such strategies with regard to AM host defense capacities during lung infection remains unknown (179, 180).

Alternative efficient approaches target AM capacity to clear pathogens via the activation of different cellular death programs. In the context of chronic obstructive pulmonary disease, an ongoing study is screening for compounds that can activate macrophage efferocytosis (ClinicalTrials.gov NCT04775394). Additionally, several studies highlighted the potential of triggering autophagy in infected AMs (181, 182). Using autophagy inducers like all-trans-retinoic acid (ATRA) helped reduce the bacterial burden in *M*. *tuberculosis*–infected AMs (183). Autophagy can also be activated by the counteracting of autophagy-inhibitory signals, such as the inhibition of mTORC1 to prevent autophagosome formation. Several mTOR inhibitors (rapamycin, everolimus) are promising in controlling the bacterial burden of *M*. *tuberculosis* infection (181).

GM-CSF, an indispensable factor for AM differentiation, survival, and homeostasis functions, improved AM host defense in bacterial and viral pneumonia models when locally deposited into the lungs of mice either by therapeutic administration or by genetic overexpression in the alveolar epithelium (63, 184–189). Intrapulmonary deposition of GM-CSF also prevented bacterial superinfection after influenza in mice (58, 190) and improved anti-influenza adaptive immunity by targeting lung-resident dendritic cells (191). Notably, GM-CSF exerts a direct effect on the injured alveolar epithelium in sterile and infectious lung injury, which improves outcome independently of the myeloid cell compartment by driving proliferation of epithelial progenitor cells and resealing the alveolar barrier (85, 192). Inhaled recombinant human GM-CSF (sargramostim, molgramostim) has recently emerged as a successful strategy for driving a beneficial AM phenotype and improving barrier function in pneumonia-associated acute respiratory distress syndrome, including COVID-19 (193–196), with the first phase II trials revealing encouraging results and a beneficial safety profile (ClinicalTrials.gov NCT02595060, NCT04569877). On the other hand, blocking the GM-CSF receptor with mavrilimumab in cases of severe lung damage due to late-stage COVID-19 was suggested to reduce inflammation and control lung injury (197, 198), highlighting the dual role of the GM-CSF signaling pathway in tailoring macrophage polarization within disease progression. In summary, several approaches demonstrated the potential of AMs for cell-based therapy. The aforementioned strategies highlight encouraging proof-of-concept studies, laying the foundation for further investigation of the promising potential of AMs in clinical applications.

## Concluding remarks

**Figure 2:**
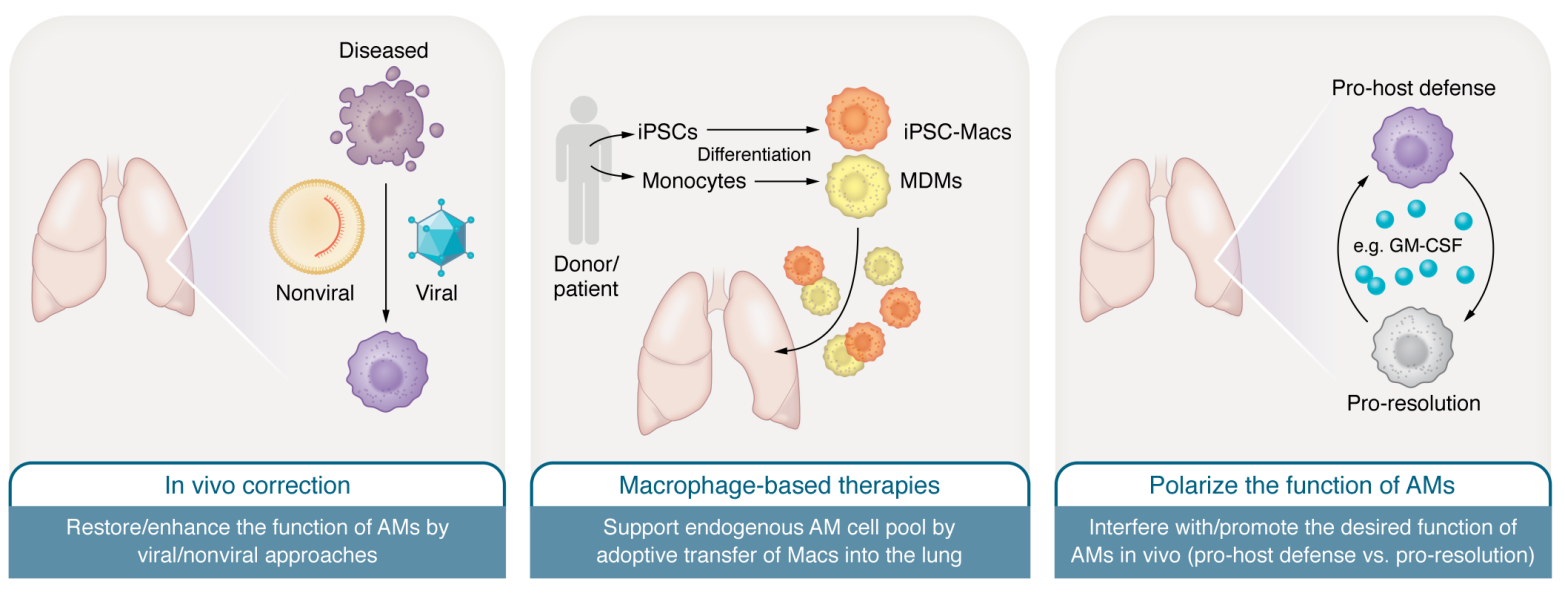
AM-targeting strategies for the treatment of pulmonary diseases. Different approaches to support and/or repair the pool of endogenous AMs are pursued. AMs can either be targeted by viral and nonviral approaches to restore and/or enhance their function in vivo (left), or be modified by different molecules to influence and/or promote a certain stage of activation to promote lung tissue repair or antibacterial function (right). An alternative and innovative approach would be the direct transfer of macrophages into the lungs of patients (middle). Suitable macrophages can be derived from human iPSCs (iMacs) or classical monocytes (monocyte-derived macrophages [MDMs]) by new immune cell farming strategies.

Specific AM-targeting or AM-based cell therapies are still in their infancy, but several promising and clinically effective strategies are emerging (Figure 2). Such diverse targeting strategies, however, pose a challenge in identifying whether the current disease stage necessitates a harnessed, suppressed, or disease-specifically modified AM. Therefore, a deep understanding of the given disease pathology, together with proper identification of inflammation onset and the disease-specific course of AM reprogramming, is needed. An increasing knowledge of the mechanistic regulations of AM functions, together with the possibility of generating stem cell–derived human macrophages, might provide therapeutic options to promote lung repair without impairing pathogen control.

## Author contributions

CM, NL, UM, and SH conceptualized the project. CM and SA performed the investigation. NL, UM, and SH acquired resources for the project. CM and SMA wrote the original draft of the manuscript. CM, SMA, NL, UM, and SH reviewed and edited the manuscript. SMA and NL visualized the project. NL, UM, and SH acquired funding for the project. All authors contributed to the article and approved the submitted version. The order of the co-first authors was decided based on responsibility for covering different manuscript aspects (introduction, main text, concluding remarks, figures, references) as well as different stages of manuscript preparation (manuscript writing and editing, coordination between the 2 sites).

## Version 1 10/02/2023

Electronic publication
